# Nanomaterials and Nanotechnology for Energy Conversion and Storage

**DOI:** 10.3390/nano14191610

**Published:** 2024-10-08

**Authors:** Chuanyin Xiong, Qiusheng Zhou

**Affiliations:** College of Bioresources Chemical & Materials Engineering, Shaanxi University of Science and Technology, Xi’an 710021, China

## 1. Introduction

The world is undergoing a new round of energy reform, and traditional fossil fuels have sparked people’s thinking due to their environmental and non-renewable issues [1,2,3]. Seeking a sustainable energy source has become a focus of attention [4,5,6]. Among them, the new battery technology based on electrochemical performance has become a possible option, and the preparation of electrode materials has become the key to the development of this field [7,8,9,10]. Researchers have designed and fabricated electrode materials through various construction methods in the hope of making contributions to this field [11,12,13,14]. Among them, regulating the distribution and modification of nanomaterials on composite materials to obtain specific functions has become a commonly used method. The renewability and degradability of biomass materials have become ideal substrates for constructing possible electrode materials [15,16,17,18]. However, compared with traditional electrode materials, biomass-based electrode materials still have significant shortcomings that need further research.

## 2. An Overview of Published Articles

Olga Boytsova et al. (Contribution 1) successfully prepared highly oriented TiO_2_ nanoparticle arrays at 450 °C using NH_4_TiOF_3_ as the raw material and polyethylene glycol-400 as the auxiliary agent and confirmed for the first time that the transition from mesoporous rutile phase to rutile phase occurs between 1000 °C and 1200 °C. The small amount of K-phase nanowhiskers generated at 800 °C increased the photocatalytic performance by four times. The TiO_2_-layered nanomaterials obtained provide prospects for the application of catalysis and electrode materials.

Ren et al. (Contribution 2) constructed a lithium–sulfur (Li-S) battery using paper-based electrodes and investigated the effects of different functionalized carbon nanotubes as conductive fibers on the physical and electrochemical properties of paper-based sulfur cathodes in lithium sulfur batteries. Among them, OH-CNT can establish a uniform and stable 3D network, exhibiting good mechanical properties and helping to reduce Li_2_S aggregation while maintaining electrode porosity. Graphitized hydroxylated carbon nanotubes (G-CNT) exhibit significant capacity at low currents due to their excellent conductivity and interaction with lithium polysulfides (LiPS). The paper-based electrode integrated with OH-CNT demonstrates good sulfur utilization efficiency and rate capacity, demonstrating the high-performance potential of lithium sulfur batteries.

Shen et al. (Contribution 3) introduced and reported the nitrogen nucleotide coordination self-assembly strategy of the metal/nitrogen-doped carbon single-atom catalyst (M-N-C SAC), which precisely adjusts the dispersion of metal atoms during the pyrolysis process to eliminate the porous carbon microspheres generated by Zn, maximize the exposure of Co-N_4_ sites, and promote charge transfer during the oxygen reduction reaction (ORR) process by controlling the metal ratio. Meanwhile, the zinc–air battery (ZAB) assembled using CoSA/N-PCMS has better power density and capacity than Pt/C+RuO_2_-based ZAB and has good practical application prospects.

Li et al. (Contribution 4) alleviated the problems of volume effect and solid electrolyte membrane (SEI) instability by constructing a core–shell structure in which silicon nanoparticles (SiNPs) were used as the core, nano-titanium dioxide (TiO_2_) was used as the buffer layer, and silver nanowires (AgNW) were doped to form a conductive network structure to improve the conductivity of the material. Made by SiNPs@TiO_2_/Compared with pure SiNPs electrode, AgNWs electrode material, SiNPs@TiO_2_/AgNWs electrodes exhibit excellent electrochemical performance, providing a new approach for the preparation of high-performance silicon-based negative electrode materials for lithium-ion batteries.

Afaq Ullah Khan et al. (Contribution 5) prepared CuSe-TiO_2_-GO composite materials using hydrothermal synthesis and found through comparison that CuSe, TiO_2_, and binary CuSe-TiO_2_ composites exhibited higher degradation efficiency compared to ternary CuSe-TiO_2_-GO composites. Among them, the conduction band of CuSe had high positivity, which led to rapid electron excitation to the conduction band of CuSe, thereby improving the photocatalytic activity of CuSe-TiO_2_-GO ternary composite materials. The holes left by graphene oxide (GO) on the surface of the MB photocatalyst, combined with the electron hole pairs assisted by GO photoexcitation, led to enhanced photocatalytic performance. In addition, the CuSe-TiO_2_-GO composite, as an electrode material for supercapacitors, also exhibits excellent capacitance performance.

## 3. Conclusions

In summary, the contribution of this Special Issue focuses on the synthesis of catalytic materials and their applications in the field of electrode materials. The main issues considered are based on energy and environmental challenges. The construction of these catalytic materials provides possible methods for the advancement of energy and investment in nanoscience and technology in sustainable energy. In addition, the focus of developing electrode materials is also based on the application and assembly of nanomaterials.

The large-scale application of catalytic materials and electrode material preparation plays an important role in solving the current energy crisis and environmental problems. Among them, the preparation of biomass electrode materials can solve the problems of material non-degradation and non-sustainability in the electrode material preparation process, which has important prospects for achieving industrial scale and green application of the entire industry chain. The possible solutions to energy and environmental challenges considered in this Special Issue have a positive impact on modern and next-generation society.
